# Integrating cortico-limbic-basal ganglia architectures for learning model-based and model-free navigation strategies

**DOI:** 10.3389/fnbeh.2012.00079

**Published:** 2012-11-27

**Authors:** Mehdi Khamassi, Mark D. Humphries

**Affiliations:** ^1^Institut des Systèmes Intelligents et de Robotique, Université Pierre et Marie CurieParis, France; ^2^Centre National de la Recherche Scientifique, UMR7222Paris, France; ^3^Department d'Etudes Cognitives, Group for Neural Theory, Ecole Normale SuperieureParis, France; ^4^Faculty of Life Sciences, University of ManchesterManchester, UK

**Keywords:** reinforcement learning, habit, stimulus-response, action-outcome, nucleus accumbens

## Abstract

Behavior in spatial navigation is often organized into map-based (place-driven) vs. map-free (cue-driven) strategies; behavior in operant conditioning research is often organized into goal-directed vs. habitual strategies. Here we attempt to unify the two. We review one powerful theory for distinct forms of learning during instrumental conditioning, namely model-based (maintaining a representation of the world) and model-free (reacting to immediate stimuli) learning algorithms. We extend these lines of argument to propose an alternative taxonomy for spatial navigation, showing how various previously identified strategies can be distinguished as “model-based” or “model-free” depending on the usage of information and not on the type of information (e.g., cue vs. place). We argue that identifying “model-free” learning with dorsolateral striatum and “model-based” learning with dorsomedial striatum could reconcile numerous conflicting results in the spatial navigation literature. From this perspective, we further propose that the ventral striatum plays key roles in the model-building process. We propose that the core of the ventral striatum is positioned to learn the probability of action selection for every transition between states of the world. We further review suggestions that the ventral striatal core and shell are positioned to act as “critics” contributing to the computation of a reward prediction error for model-free and model-based systems, respectively.

## 1. Introduction

A vast morass of neuroscience data addresses the problem of how voluntary behavior is underpinned by the anatomical and physiological substrates of the forebrain. Principles or frameworks to organize this data are essential. A consensus is growing around the potentially useful organizing principle that we can make a division of the forebrain striatum into three domains on both anatomical (Joel and Weiner, 1994, 2000; Voorn et al., 2004) and functional (Yin and Knowlton, 2006; Yin et al., 2008; Bornstein and Daw, 2011; Ito and Doya, 2011; van der Meer et al., 2012) grounds. From this “striatal eye-view” we can make sense of the wider cortical, hippocampal, amygdala, and basal ganglia networks in which they sit, and the role of these networks in different forms of voluntary behavior. Both the spatial navigation and instrumental conditioning literatures have adopted this perspective, recognizing the functional division of striatum into dorso-lateral (DLS), dorso-medial (DMS), and ventral striatum (VS)^1^, belonging to different parallel cortico-basal ganglia loops (Alexander et al., 1990; Middleton and Strick, 2000), with each striatal domain having established functional roles within those broader behavioral distinctions. How do these functional distinctions map between the two literatures? And what might we learn by comparing the two?

While some links have been drawn between the approaches of the two literatures (Redish, 1999; Yin et al., 2004, 2008; Khamassi, 2007), their primary theories for the strategies underpinning behavior are, we suggest, orthogonal: the conditioning literature distinguishes *goal-directed* and *habitual* behavior in a task, whereas the navigation literature distinguishes *place* and *response* strategies for solving a task. However, there is mounting evidence that the place/response distinction is unable to account for the effects of lesions on navigation behavior. Our main hypothesis is that strategies for navigation, similar to strategies for instrumental conditioning (Daw et al., 2005), can be reconciled as either *model-free* or *model-based*—we define these terms below. At root, the key distinction is that it is the *use* of information in building a representation of the world, rather than the *type* of information about the world, that defines the different computational processes and their substrates in the striatum. We argue that explicitly identifying the DLS as a central substrate for model-free learning and expression, and the DMS as a central substrate for model-based learning and expression (Yin and Knowlton, 2006; Thorn et al., 2010; Bornstein and Daw, 2011; van der Meer et al., 2012) can help reconcile numerous conflicting results in the spatial navigation literature.

With this hypothesis in hand, we can see how work on spatial navigation gives us a second hypothesis, useful to understanding instrumental conditioning. We propose that the VS is a central substrate—in collaboration with the hippocampus—for a collection of functions that we informally term the “model-builder”. On the one hand, the core of the VS acting as the locus of actions necessary to build a model; and on the other hand the shell of the VS acting to evaluate predicted and achieved outcomes in the model. These are clearly not the only roles of the multi-faceted VS (Humphries and Prescott, 2010); nonetheless, they may prove a further useful organizing principle.

With this sketch in mind, we address first the different forms of behavioral strategies that have separately been identified in the spatial navigation and instrumental conditioning literatures. We take a striatal-centric view here as an organizing principle, not as a claim that striatal domains are exclusive substrates for different forms of learning and navigation. Each striatal domain is one locus in a broader basal ganglia network that computes its output using information gathered by the striatum (Houk and Wise, 1995; Mink, 1996; Redgrave et al., 1999; Humphries et al., 2006; Leblois et al., 2006; Girard et al., 2008); and each network is in turn one locus in a broader basal ganglia-thalamo-cortical loop. Nonetheless, the striatum's consistent intrinsic microcircuit across the dorsolateral to ventro-medial axis (Bolam et al., 2006), its integration of cortical, thalamic, hippocampal, and amygdala input, and its position as the primary target of the midbrain dopaminergic system, makes it a natural vantage point from which to attempt to unify the disparate strands of navigation and conditioning.

## 2. Strategy distinctions in spatial navigation

### 2.1. Taxonomy of spatial navigation forms

Evidence for different navigation strategies in the rat comes from behavioral studies showing that they are able to rely on different information to localize themselves in the environment and to reach a certain location in space (Krech, 1932; Reynolds et al., 1957; O'Keefe and Nadel, 1978). Existing classifications of navigation strategies (O'Keefe and Nadel, 1978; Gallistel, 1990; Trullier et al., 1997; Redish, 1999; Franz and Mallot, 2000; Arleo and Rondi-Reig, 2007) point out a series of criteria, some of them overlapping, to differentiate navigation strategies: the type of information required (sensory, proprioceptive, internal), the reference frame (egocentric vs. allocentric), the type of memory at stake (procedural vs. declarative memory) and the time necessary to acquire each strategy (place-based strategies generally being more rapidly acquired than cue-guided strategies; Honzik, 1936; O'Keefe and Nadel, 1978; Packard and McGaugh, 1992, 1996; Redish, 1999). Moreover, it has been observed that in normal animals, a shift from a place strategy to a response strategy occurs in the course of training (Packard, 1999). This has led to the proposition of a strong distinction between two main categories of strategies:

- *Response* strategies, where a reactive behavior results from learning direct sensory-motor associations (like heading toward a visual cue or making an egocentric turn at the crossroads of a maze). This category includes target-approaching, guidance, cue-guided, and praxic^2^ normally refers to internally-generated sequences of movement independent of position information. navigation, and can be further elaborated in the form of a sequence or chaining of Stimulus-Response (S-R) associations when new cues result from the previous displacement (O'Keefe and Nadel, 1978; Trullier et al., 1997; Arleo and Rondi-Reig, 2007).
- *Place* strategies, which rely on a spatial localization process, and can imply a topological or metric map of the environment (Tolman, 1948)—the term *map* being defined by Gallistel (1990) as “a record in the central nervous system of macroscopic geometric relations among surfaces in the environment used to plan movements through the environment”.

### 2.2. Substrates in the striatum

This strong strategy distinction has been mapped onto a strong distinction in underlying neural systems. It has been found that lesions of the hippocampal system impair place strategies while sparing response strategies (Morris, 1981; Packard et al., 1989; Devan and White, 1999). In contrast, lesions of the DLS produce the opposite effect: impairing or reducing the expression of response strategies while sparing place strategies (Potegal, 1972; Devan and White, 1999; Adams et al., 2001; Packard and Knowlton, 2002; Martel et al., 2007). Thus, it is common to speak of place and response strategies as being, respectively, “hippocampus-dependent” and “hippocampus-independent” (White and McDonald, 2002). Some theories propose that the “hippocampus-dependent” system expresses its output via the VS (Redish and Touretzky, 1997; Albertin et al., 2000; Arleo and Gerstner, 2000; Johnson and Redish, 2007; Penner and Mizumori, 2012). Other studies have also highlighted a role for the DMS in the “hippocampus-dependent” system (Whishaw et al., 1987; Devan and White, 1999; Yin and Knowlton, 2004), by finding that lesions of the DMS promote response strategies, implying the loss of place strategies. The behavioral strategies are often equated directly with learning systems: that is, separate systems that learn a particular cue-guided and/or place-guided set of strategies for a given environment. However, the simple mapping between VS-DMS vs. DLS onto place vs. response strategies is not consistent with mounting evidence from lesion studies.

### 2.3. Known problems with taxonomy and substrates

Response strategies are not solely dependent on the DLS. Chang and Gold (2004) reported that DLS-lesioned rats were only unable to express a response strategy on a T-maze in the absence of extra-maze cues; in cue-rich conditions the DLS-lesioned rats did not differ from controls in their ratio of using response or place strategies. Both Yin and Knowlton (2004) and De Leonibus et al. (2011) also found no significant decrease in the use of response strategies by DLS-lesioned rats running a T-maze. Moreover, Botreau and Gisquet-Verrier (2010) not only replicated this result but also ran a second separate cohort of DLS-lesioned rats to confirm it; further, they showed that the DLS-lesioned rats using a response strategy were really doing so: they continued to use that strategy to solve a new task on the T-maze. We conclude that the *response* learning system—including *cue-guided* and *praxic* strategies—cannot be simply associated with the DLS.

Place strategies are not solely dependent on the DMS. When learning to navigate to a hidden platform in the Morris water maze, rats with DMS lesions were able to learn the platform's location just as well as controls or DLS-lesioned rats, as indicated by their similar escape latencies (Whishaw et al., 1987; Devan and White, 1999); consistent impairment—shown by a lack of improvement over trials—only occurred if the fornix-fimbria^3^ was cut (Devan and White, 1999). Botreau and Gisquet-Verrier (2010) reported that DMS-lesioned rats did not differ from controls or DLS-lesioned rats in their ratio of using response and place strategies in a probe test in the water-maze. We conclude that the * place* learning system cannot be simply associated with the DMS.

The precise role of VS in particular navigation strategies is even less clear (see Humphries and Prescott, 2010; Penner and Mizumori, 2012 for recent reviews). VS lesions impair place-based learning (Sutherland and Rodriguez, 1989; Ploeger et al., 1994; Setlow and McGaugh, 1998; Albertin et al., 2000). For instance, lesions of the medial shell of the VS impair the rat in learning and recalling the location of sites associated with larger rewards (Albertin et al., 2000). However, more recent studies reveal that VS function may not be restricted to place strategies. For instance, De Leonibus et al. (2005) report that VS lesions impair the acquisition of both allocentric and egocentric strategies in a task requiring the detection of a spatial change in the configuration of four objects placed in an arena.

The clean distinction between rapidly learnt place strategies and slowly learnt response strategies is also problematic. Several authors have reported rapidly learned response strategies (Pych et al., 2005; see Willingham (1998) and Hartley and Burgess (2005) for reviews including rodent data). Conversely, while place strategies have most of the time been found highly flexible and more rapidly acquired than response strategies (Packard and McGaugh, 1996), after extensive training place strategies can also become inflexible and persist in leading animals toward the previous goal location after a reversal, as if not relying on a cognitive map (Hannesson and Skelton, 1998; see also rat behavioral data in a Y-maze described in Khamassi, 2007).

These data suggest that the simple distinction between place vs. response strategies might be too broad to explain the different roles of VS-DMS vs. DLS in navigation. Several authors have highlighted that this classification of navigation strategies lends too much importance to the *type* of information involved (i.e., place vs. cue) and thus to the spatial localization process (Trullier et al., 1997; Sutherland and Hamilton, 2004). We suggest that considering the type of learning involved—and measurable in terms of behavioral flexibility—might better account for the specific involvement of VS, DMS, or DLS in navigation. To see this, let us first consider the taxonomy of learning in instrumental conditioning.

## 3. Strategy distinctions in instrumental conditioning

### 3.1. Goal-directed behaviors vs. habits

A long line of conditioning research has elaborated two operationally defined forms of instrumental behavior in the rat: *goal-directed* in which the animal is able to modify its behavior in response to changes in outcome and *habitual* in which the animal does not respond to changes in outcome (it perseveres with its previous action— hence “habit”) (Dickinson, 1985; Yin et al., 2008). This definition is “operational” because it can only be safely defined in retrospect—i.e., after extinction. Experimenters typically use a test in extinction to discriminate between these two behavioral modes after a reward devaluation or change in contingency between behavior and reward. If during this extinction test the animal quickly stops producing the now irrelevant conditioned response (e.g., pressing a lever) it is said to be *goal-directed*; if the animal persists it is said to be *habitual* (Balleine and Dickinson, 1998). The inference is then drawn that goal-directed animals have access to action-outcome contingencies to guide behavioral choice, and that changes in outcome consequently change action choice, whereas habitual animals make behavioral choices based on S-R pairings (Dickinson, 1985).

### 3.2. Substrate evidence for DMS' goal-directed and DLS' habitual roles in learning

During the course of a conditioning task animals' behavior progressively shifts from expressing awareness of action-outcome contingencies to expressing habits. In particular, after extensive training or *overtraining* animals' behavior is most often habitual (Yin et al., 2004). It turns out that this natural progressive shift can be perturbed by lesions of different parts of the striatum, pointing to a possible double-dissociation between DLS and DMS: the former being required for acquisition and maintenance of habits, and the latter being required for learning and expression of goal-directed behaviors (Balleine, 2005; Yin and Knowlton, 2006; Yin et al., 2008).

There is a strong consensus that the dorsolateral striatum is necessary for habitual behavior: lesions of either the DLS (Yin et al., 2004), or disruption of dopamine signaling within it (Faure et al., 2005), prevent habit formation in extinction. Animals with such lesions thus appear to maintain goal-directed behavior throughout a task. Correspondingly, there is a re-organization of the DLS' single neuron activity during habit formation (Barnes et al., 2005; Tang et al., 2007; Kimchi et al., 2009). Consequently, the dorsolateral striatum has been proposed as central to the learning of habits (Yin and Knowlton, 2006; Yin et al., 2008).

There is a strong consensus that the dorsomedial striatum is necessary for goal-directed behavior: lesions of the DMS (Yin et al., 2005b), or blockade of NMDA receptors within it (Yin et al., 2005a), putatively preventing synaptic plasticity, prevent sensitivity to devaluation or contingency changes in extinction. Animals with such lesions thus appear to obtain habitual behavior from the outset. Correspondingly, there is a re-organization of the DMS' single neuron activity after changes in action-outcome contingencies (Kimchi and Laubach, 2009; Kimchi et al., 2009). Consequently, the dorsomedial striatum has been proposed as central to goal-directed learning (Yin and Knowlton, 2006; Yin et al., 2008).

A caveat is that the anterior part of DMS (aDMS) may escape from this functional scheme. To our knowledge, only the posterior DMS (pDMS) has been clearly shown as involved in the acquisition of goal-directed behaviors (Yin et al., 2005b) and in place-based navigation (Yin and Knowlton, 2004). Lesions of aDMS do not affect either of these processes. They even increase the number of rats classified as place-responders both during initial and late phases of learning (Yin and Knowlton, 2004), and seem to increase the sensitivity to contingency degradation (compared to sham-lesioned rats) (Yin et al., 2005b). Ragozzino and Choi (2004) showed that inactivating aDMS does not affect learning of a T-maze task or acquisition of a place strategy; but inactivation during reversal learning did affect performance, thus suggesting that aDMS is involved in switching between strategies, not in learning *per se*. Contrary to these data, Moussa et al. (2011) showed that a rat's impairment in learning an alternating-arm T-maze task correlated with volume of DMS damage, not with the location of the lesion. Nonetheless, it remains possible that the aDMS is not part of the goal-directed or habitual systems.

### 3.3. The ventral striatum in conditioning

While dorsal parts of the striatum are important for the expression of learned S-R contingencies, their acquisition may require intact VS (Atallah et al., 2007). The VS is indeed located at a crossroads between limbic and motor structures which places it in a privileged position to integrate reward, motivation, and action (Mogenson et al., 1980; Groenewegen et al., 1996). In the instrumental conditioning literature, the VS is also considered particularly important for Pavlovian influences over voluntary behavior (Balleine and Killcross, 1994; Dayan and Balleine, 2002; Yin et al., 2008; van der Meer and Redish, 2011). It has been attributed roles as both a locus of Pavlovian conditioning—learning to associate outcomes to different stimuli or states—and the locus of Pavlovian-instrumental transfer—the use of those learnt stimulus-outcome associations to motivate the learning and expression of instrumental actions in the presence of those stimuli (Yin et al., 2008). Further, while the functional subdivision of VS into core and shell might be oversimplified (Heimer et al., 1997; Ikemoto, 2002; Voorn et al., 2004; Humphries and Prescott, 2010), it may account for distinct influences of reward values on habitual performance and goal-directed behavior, respectively. For instance, Corbit and Balleine (2011) found that shell lesions impair outcome-specific [putatively goal-directed as noted by Bornstein and Daw (2011)] Pavlovian-instrumental transfer while core lesions impair general (putatively habitual) Pavlovian-instrumental transfer.

These data suggest that the differences in the learning process controlling the progressive influence of rewards on actions may determine the functional roles of striatal domains in various behavioral strategies: DLS being involved in learning and expression of habitual behaviors; DMS being involved in learning and expression of goal-directed behaviors; VS controlling the influence of reward values on these two processes during learning. Computational work has brought great advances in formalizing the differences between these learning processes.

### 3.4. Model-based vs. model-free learning processes

**Figure 1:**
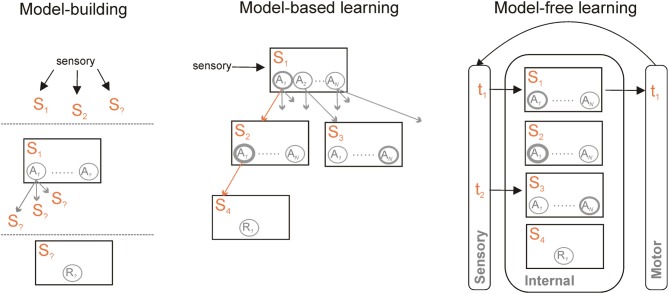
**Model-based and model-free learning and controllers.** Model-based and model-free controllers represent the world as a set of states *S*_1_ … *S*_*m*_ and actions *A*_1_ … *A*_*N*_ within those states. They learn the values of each action in a given state, here indicated by the thickness of each circle, based on available rewards *R*. What distinguishes them is their representation of the links between those states. A model-based controller **(centre)** also represents the transitions between states and the action(s) that cause the transition (indicated by the multiple arrows). For a known current state, specified by current sensory information, the model can be traversed to find the likely outcome of simulated actions in each state—one such trajectory is given by the orange arrows. Each trajectory can then be used to update the predicted value of each action. Finally, after a number of trajectories through the model, an overt action is selected based on their updated values in the current state. A model-free controller **(right)** vastly reduces the representational and computational demands by essentially externalizing the world-model. Sensory information specifies the state at time *t*_1_; an action is chosen based on its current value. Updated sensory information resulting from that action then specifies the state at time *t*_2_. Learning is then based on the prediction error between expected and resulting values of the action taken at *t*_1_. A model-free controller can also be trained by a model-based controller, and thus represent an abstraction of that model. Irrespective of whether model-free or model-based, a common set of information needs to be learnt to construct and use the controller **(left)** to specify the set of current relevant states in the world; to learn actions available within them and the transitions those actions cause; and to learn the reward function—which state(s) contain reward(s).

Machine-learning research into formal algorithms for reinforcement learning has developed a basic distinction between two forms of such algorithms. Common to both is the idea that we can represent the world as a set of states 𝕊, that the agent could take one of a set of actions 𝔸 in each state (including no action at all), and that the outcome of taking action *a* in state *s* is the next state *s*′ and a possible reward *r* (Sutton and Barto, 1998). Distinguishing the two is whether or not the dependencies in the world representation are explicitly modeled (Figure 1).

In the *model-free* forms of algorithm, each state has associated with it a distribution of the values of each possible action, learnt iteratively using a prediction error to minimize the difference between the values of actions in consecutive states. This set includes most well-known forms of reinforcement learning algorithms—including Temporal Difference (TD) learning, Actor-Critic, and Q-Learning. Each state thus has an associated distribution of cached action-values *Q*(*s*, *a*) over all available actions. The action to execute is then simply chosen based on this cached value distribution. Such behavior is called reactive in that it is state-driven—e.g., stimulus-driven—and does not rely on the inference of possible outcomes of the action.

In the *model-based* forms of algorithm, direct use is made of the state information about the world. With each state *s* is still associated a reward *r*, each action is still assigned a value *Q*(*s*, *a*), and action selection is based on those values. However, model-based algorithms explicitly store the state transitions after each action: they can then simulate off-line the consequence of action choices on transitions between states before choosing the next action appropriately (Sutton and Barto, 1998; Johnson and Redish, 2005). Thus in this case the agent will infer possible future outcomes of its decisions before acting. In simple decision-making tasks in which each action leads to a different state, such a process is naturally captured by a branching decision tree (Figure 1); in more natural situations states may be re-visited during ongoing behavior, and thus the transitions between states may have periodic structure. Sophisticated model-based algorithms explicitly compute a separate transition matrix *T*(s′, *a*, *s*) for the probability of ending up in each next state *s*′, given the current state *s* and each possible action choice *a* in 𝔸 (Daw et al., 2005, 2011; Glascher et al., 2010).

Daw et al. (2005) proposed the formal mapping that goal-directed behavior results from model-based learning and that habitual behavior results from model-free learning^4^. They further proposed that both learning systems operate in parallel, with the system chosen for current behavioral control based on having the least uncertainty in its prediction of the outcome. Using stylized examples of simple conditioning tasks, they showed how this mapping can explain the sensitivity to devaluation and contingency degradation in extinction early in training when the model-based controller is dominant, and how that sensitivity is lost when the model-free controller becomes dominant with over-training. The underlying explanation is that the model-based controller directly represents action-outcome contingencies, and is thus able to quickly propagate changes in reward through the world-model; by contrast, the model-free controller, while able to reduce the uncertainty in its predictions with over-training, requires further extensive training for the change in reward to propagate through the independent state-action representations. This formal mapping onto computational substrates has proven a very useful and fruitful guide to the understanding of these operationally-defined forms of behavior and their inferred learning systems (Ito and Doya, 2011; Bornstein and Daw, 2011; van der Meer et al., 2012).

This computational mapping is also assumed to follow the same substrate mapping (Daw et al., 2005; Bornstein and Daw, 2011; Ito and Doya, 2011). Thus, as DLS is central to the habit-learning system, so, by extension, it is considered central to the model-free learning system in instrumental conditioning (Daw et al., 2005). Similarly, as DMS is central to the goal-directed system, it is thus natural to propose that DMS is central to the model-based learning system in instrumental conditioning (Bornstein and Daw, 2011).

## 4. Unification: navigation strategies are model-free or model-based

Superficially, the model-free/model-based dichotomy strongly resembles the dichotomous taxonomy defined in the spatial navigation literature between flexible map-based *place* strategies and automatic map-free *response* strategies. However, the two approaches are orthogonal: one is defined by information use in a world representation (model-free/based), the other by information type (place/cue).

**Figure 2:**
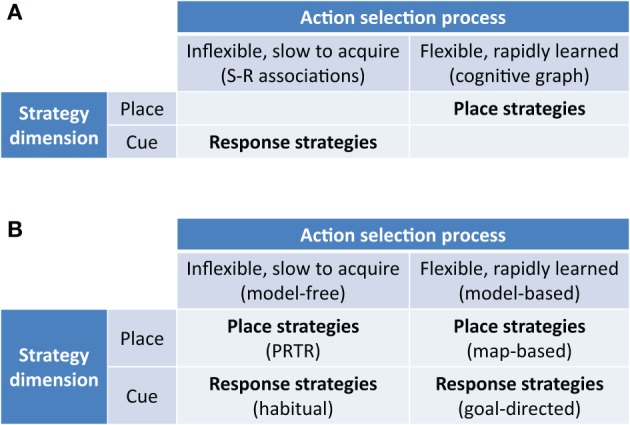
**New taxonomy of navigation strategies based on model-based/model-free reinforcement learning. (A)** Previous taxonomies highlight the distinction between flexible rapidly acquired map-based strategies and inflexible slowly acquired S-R strategies. **(B)** New taxonomy highlighting model-free and model-based place strategies as well as model-free and model-based response strategies. PRTR, place-recognition triggered response strategies as classified by Trullier et al. (1997).

Our hypothesis is that we may similarly distinguish model-free and model-based navigation strategies by their use of information (Figure 2), no matter if the state is represented by a spatial location or a visual stimulus. Within these two top-level strategies, we may further differentiate strategies defined by their reference frame and modality of processed stimuli:

- egocentric reference frame, relying on idiothetic (praxic), or allothetic (cue-guided) stimuli;
- allocentric reference frame, relying on idiothetic and/or allothetic stimuli (places).

Our hypothesis thus naturally extends to proposals for the striatal substrates of model-free and model-based strategies in navigation: that the DLS is central to the model-free navigation system and DMS is central to the model-based navigation system.

This combined conceptual (model-free vs. model-based) and substrate (DLS vs DMS) hypothesis raises four implications that each explain some troubling or inconsistent data for the place vs. response dichotomy in navigation. First, that we can conceive of a model-free strategy based on place information alone supported by the DLS. Second, that, correspondingly, we can conceive of a model-based “response” strategy based on cues alone supported by the DMS. Third, that, following the model-based/model-free mapping in conditioning (Daw et al., 2005), model-based and model-free control of navigation could be distinguished behaviorally by whether or not the animal reacts to changes in the value or contingencies of rewards, and by lesions to the DLS and DMS. Fourth, that both place and cue information should be available to both the model-based and model-free navigation systems, and thus should be detectable within both the DMS and DLS. We consider each of these in turn, then discuss the key role of the hippocampal formation as the likely source of state information.

### 4.1. DLS and (model-free) place strategies

Model-free navigation strategies based on place information alone have been called “Place-Recognition Triggered Response (PRTR)” strategies by Trullier et al. (1997) who emphasized that such a strategy produces inflexible behavior because it needs to re-learn sequences of place-response associations in case of a change in goal location. This type of learning was prominent in early models of hippocampus-dependent navigation (Burgess et al., 1994; Brown and Sharp, 1995; Arleo and Gerstner, 2000; Foster et al., 2000).

Following the same DLS vs. DMS double-dissociation logic as was used for goal-directed and habitual learning then, if DMS is the substrate for place strategies, lesions of the DMS should impair place strategies and lesions of the DLS should not affect them. However, there is evidence against this dissociation and indirect evidence in favor of a place strategy supported by DLS. Lesions of the DMS slow but do not prevent the learning of a hidden platform in a water maze, which putatively requires a place-based strategy (Devan and White, 1999). More compelling, Botreau and Gisquet-Verrier (2010) tested control, DLS-lesioned, and DMS-lesioned rats learning a hidden platform water maze task; after learning, a probe trial was used where the rats were started in a different location for the first time: they found that rats were divided into the same ratio of “place” and “response” groups on the probe trial irrespective of whether they were control, DLS-lesioned, or DMS-lesioned rats. Recently, Jacobson et al. (2012) tested rats on an alternating strategy plus-maze, which required the use of either a response-based or place-based strategy on each trial as signaled by an extra-maze cue: they found that post-training DLS lesions impaired use of both the response and place strategies. Thus, there is evidence that intact DLS is important for using place strategies.

### 4.2. DMS and (model-based) response strategies

The proposal of a model-based response strategy is just the claim that we can conceive of states in a spatial navigation task as being defined by the position of intra- or extra-maze cues relative to the animal. In such a model, different states would not necessarily correspond to different spatial position. Rather, we can conceive of an example task where distinct states *s*_1_ and *s*_2_ correspond to the same spatial location and differ on whether a light is turned on or off. Then a model-based system can learn the transitions between these states and search the model to proceed with action selection—e.g., reward may be delivered only when the light is on. Thus, whereas others have explicitly identified a response strategy—e.g., a strategy guided by the light—with habitual behavior (e.g., Yin and Knowlton, 2004), we are proposing that the two are orthogonal.

Again we may follow the same double-dissociation logic: if DLS is the sole substrate for response strategies, then lesions of the DLS should impair response strategies and lesions of the DMS should not affect them. There is evidence against this dissociation, and in favor of DMS involvement in response-strategies. As noted in section 2.3, lesions of the DLS do not impair the use of response strategies on probe trials, suggesting that intact DMS is sufficient to support the use of response strategies (Chang and Gold, 2004; Yin and Knowlton, 2004; Botreau and Gisquet-Verrier, 2010; De Leonibus et al., 2011). Chang and Gold (2004) further reported that the DLS lesions only effectively impaired the use of response strategies when there were no extra-maze cues. This suggests that model-based (and putatively DMS-based) use of cues was sufficient to maintain a response strategy in the cue-rich conditions; but that a model-free (and putatively DLS-based) praxic response strategy was necessary in the cue-deficient conditions (that is, in the absence of sufficient cues, learning a sequence of turns was required).

Moussa et al. (2011) tested the effects of DLS and DMS lesions on the ability of rats to learn a return-arm T-maze in which the rats were required to alternate their choice of visited arm (left or right) to obtain reward, but were free to run at their own pace. The task is a seemingly simple response strategy but requires a minimal model to achieve rewards above chance level. At the choice point of the T-maze, a model-free learning system would assign equal value to turning left or turning right as both would be rewarded on (approximately) half the visits. To achieve better, a minimal model would be needed to at least link the previous choice of arm to the current choice, chaining at least two (state, action) pairs in a loop—which corresponds to a model-based process. Moussa et al. (2011) found that DMS lesions, and not DLS lesions, impaired learning of this task irrespective of the amount of training. Their data thus suggest a model-based response strategy role for DMS.

### 4.3. Value-sensitivity in navigation and its alteration by DMS but not DLS lesions

If the prediction of Daw et al. (2005) is correct, then model-based and model-free control of action can be distinguished behaviorally by whether or not the animal reacts to changes in the value or contingencies of rewards. Thus, under our hypothesis, such sensitivity to value or contingency changes in spatial navigation should be reflected in *both* place and response strategies if using a model-based controller and in *neither* place nor response strategy if using a model-free controller. Similar to the goal-directed to habitual transfer observed in instrumental conditioning (Yin and Knowlton, 2006), we might expect that this outcome sensitivity would disappear with over-training on a sufficiently deterministic task, reflecting the transfer from a model-based to a model-free controller for navigation. Also similarly, our hypothesis is that this transfer is from the DMS to the DLS-based systems; so lesions to those systems should differentially affect how changes in value subsequently change behavior.

Whereas above we reviewed evidence in favor of their breaking the place vs response dichotomy, here we consider evidence more directly in favor of the association of DMS with a model-based system and DLS with a model-free system. De Leonibus et al., (2011) recently provided intriguing evidence from devaluation in favor of both (1) the existence of model-based and model-free response strategies and (2) their dissociable modulation by DMS and DLS lesions. Further, Moussa et al. (2011) provided evidence from extinction during navigation for both. We consider these studies in turn.

**Figure 3:**
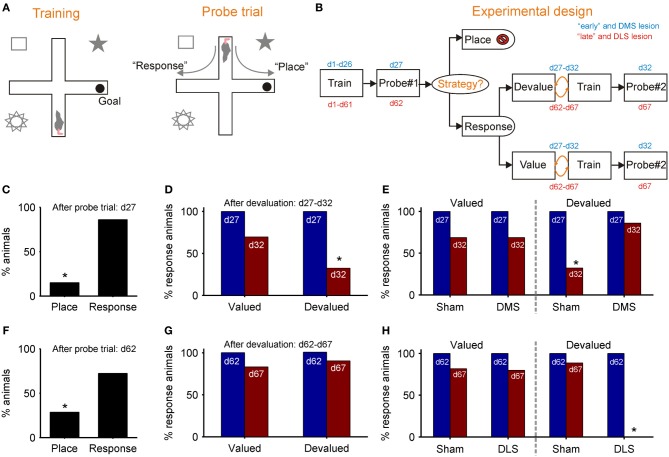
**Evidence for model-based and model-free navigation in data reported by De Leonibus et al. (2011). (A)** Dual-solution plus-maze task used by De Leonibus et al. (2011). On training trials, rats always start from the same arm (south) and have to learn the location of the reward in a consistently baited arm (e.g., east). After training, a probe trial starting in the opposite arm is used to ascertain the rat's strategy for locating the reward (a food pellet): a “response” strategy based on direction of turn, or a “place” strategy based on location of reward with respect to extra-maze cues. **(B)** The experimental design of De Leonibus et al. (2011). Rats were in two broad categories, designated “early” and “late” with respect to the first probe trial (day 27 or day 62). All “response” rats from that trial were taken forward to the second stage, and split approximately evenly into devaluation and control (value) groups. Both groups had free access to food pellet reward for 15 min immediately after training for each of five days; the devaluation group received an injection of LiCl immediately afterwards, the control group received a saline injection. The devaluation group developed a taste aversion to the pellets, but no reduction in completed trials (De Leonibus et al., 2011). **(C–E)**: data from “early” group; **(F–H)** data from the “late” group. **(C)** Proportion of “early” group rats using each strategy on first probe trial. **(D)** From the second probe trial, the proportion of rats continuing to use a “response” strategy after devaluation compared to controls. **(E)** From the second probe trial, the proportion of rats continuing to use a response strategy after devaluation and pre-training DMS lesion, compared to controls for both. **(F)** Proportion of “late” group rats using each strategy on first probe trial. **(G)** From the second probe trial, the proportion of “late” group rats continuing to use a “response” strategy after devaluation, compared to controls. **(H)** From the second probe trial, the proportion of rats continuing to use a response strategy after devaluation and pre-training DLS lesion, compared to controls for both. An * indicates a significant difference of at least *p* < 0.05—see De Leonibus et al. (2011) for details.

Figures 3A,B outlines De Leonibus et al. (2011) dual-solution plus-maze task and experimental design. Key to the design was separately training “early” and “late” groups of rats for, respectively, 26 and 61 days before the first probe trial, which established the strategy they were using to locate the reward (Figure 3B). Both “early” and “late” groups preferentially used the response strategy on the first probe trial (Figures 3C,F), replicating earlier results (Devan and White, 1999; Yin and Knowlton, 2004). However, the response strategy sub-group for both “early” and “late” were then split, with approximately half receiving a devaluation regime for the food reward in the maze. On the subsequent second probe trial, only the “early” group showed awareness of the devaluation, through a significant drop in their use of a response strategy (Figure 3D). There was no change in the use of response strategy by the devalued “late” group (Figure 3G). Thus, while both “early” and “late” groups of rats preferentially used a response strategy, only the early group modified use of that strategy after change in the value of reward, evidence of a distinction between a model-based and model-free form of response strategy.

De Leonibus et al. (2011) then separately tested the effects of pre-training sham and DMS lesions on a new “early” group, and of pre-training sham and DLS lesions on a new “late” group. They found that the DMS lesion prevented the devaluation from changing the proportion of “early” group rats using a response strategy (Figure 3E). This is consistent with the loss of DMS preventing value updates from propagating through the model-based system. Conversely, they found that the DLS lesion now permitted the devaluation to change the proportion of “late” group rats using a response strategy (Figure 3H). This is consistent with the loss of DLS preventing transfer to the model-free system, and subsequently value updates continued to propagate through the model-based system. Together, these results support the double dissociation of DMS as part of a model-based and DLS as part of a model-free system for navigation.

Moussa et al. (2011) found results consistent with this picture from rats tested in extinction on a navigation task. As noted above, they tested rats on an alternating arm T-maze task, thus requiring rats to maintain a memory of the previously visited arm. As the rats ran at their own pace, Moussa et al. (2011) were unusually also able to test the effects of extinction on navigation tasks by leaving the arms unbaited in the final 10-min session. They found that control rats did decrease their laps of the maze over the 10-min period, so that extinction effects were detectable. Moreover, though DLS lesions had no effect on learning the task, they did lead to significantly faster extinction of maze running. These data are thus consistent with lesions of DLS removing the putative model-free navigation substrate, thus leaving intact the putative model-based substrate in DMS that was subsequently faster to respond to the outcome devaluation.

### 4.4. Place and cue information is available to both model-based and model-free systems

If the DLS and DMS are indeed, respectively, substrates for model-free and model-based navigation systems, and not the response and place systems, then cue- and place-based correlates of movement should appear in the activity of both.

DLS activity is consistent with the development of cue-based correlates of movement. Jog et al. (1999) showed that developing DLS activity over the course of a T-maze task stabilized to just the start and end positions in the maze once the rats had reached operationally “habitual” behavior. van der Meer et al. (2010) showed that decoding of position information from dorsal striatal activity consistently improved over experience, and that its activity peaked only at choice points in the maze, consistent with a slow learning model-free system that learnt to associate differentiable intra-maze states with actions (Graybiel, 1998; Yin and Knowlton, 2006). DLS activity is also selectively correlated with position: Schmitzer-Torbert and Redish (2008) found that dorsolateral striatal electrophysiological activity correlated with place when the task required knowledge of spatial relationships, but no correlation when the task was non-spatial.

DMS is clearly in receipt of place information in that activity is correlated with actions or rewards in particular locations, but not correlated with the location alone (Wiener, 1993; Berke et al., 2009). Furthermore, lesions of posterior DMS prevent execution of place-based strategies (Yin and Knowlton, 2004) as does loss of dopamine from that region (Lex et al., 2011). Its input from the prefrontal cortex (PFC), particularly medial PFC which receives considerable direct input from the CA1 place cells, is one of the most likely sources of place information; there is clear evidence that medial PFC supports place representation [e.g., Hok et al. (2005)]. Nonetheless, there is also evidence for DMS' receipt of cue-information. Devan and White (1999) reported that asymmetric lesions (unilateral hippocampus and contralateral DMS) produced mild retardation of acquisition of both cue-based and place-based learning. Correspondingly, recording studies report that the largest changes in DMS neural activity occur in the middle stages of learning during cue-guided (both with auditory and tactile cues) navigation (Thorn et al., 2010).

### 4.5. Hippocampal input to model-based and model-free systems

For spatial navigation the primary candidate for generating the states and the relationship between them is the hippocampal formation. Although hippocampus has been largely associated with spatial encoding (O'Keefe and Nadel, 1978), it could be more broadly involved in learning (and planning in) a model or graph of possible transitions between states, no matter if these states are spatial or not (van der Meer et al., 2012). Consistent with this, hippocampal place cells are also sensitive to non-spatial information (e.g., the presence of a certain object or the color of the walls), this non-spatial information modulating or re-mapping the place representation (Wiener et al., 1989; Redish, 1999). Similarly, hippocampal place cells re-map on maze tasks following a change of context, such as the change of rewarded arm in a plus-maze (Smith and Mizumori, 2006). Thus, within our proposal, the role of the hippocampus would be both to supply spatial information to a model-free system and to contribute to a model-based system by building the model—in interaction with the VS as argued later—and planning actions within this model. This view is similar to ideas that the hippocampus provides contextual information to some aspects of learning such as contextual fear conditioning (Rudy, 2009) and spatial planning information to other aspects of learning (Banquet et al., 2005; Hasselmo, 2005; Dollé et al., 2010; Martinet et al., 2011). It is also similar to points made by Redish and Touretzky (1998) that one can both store sequences and do location-recall in hippocampal attractor networks without interfering with each other (see also Redish, 1999).

**Figure 4:**
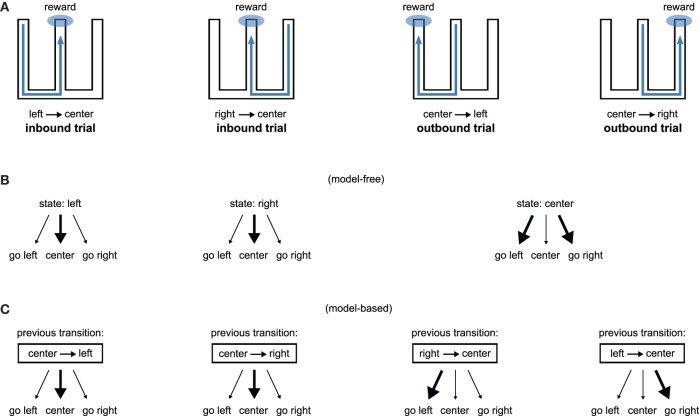
**Model-based/model-free framework applied to a spatial alternation task requiring both inbound and outbound learning. (A)** W-shaped maze experienced by rats, adapted from Kim and Frank (2009). Hippocampal lesions impair both inbound and outbound learning (Kim and Frank, 2009) while disruption of awake hippocampal replay only impairs outbound learning (Jadhav et al., 2012). **(B)** A model-free system associating places with actions can learn inbound trials but would face high uncertainty during outbound trials. **(C)** A model-based system associating previous transitions with actions can associate past experience with current location and is thus able to learn both inbound and outbound trials.

Consequently, lesions of the hippocampus should affect both model-free and model-based systems through loss of spatial information, but transient interference with its activity should affect only the model-based system through loss of the use of the model. Figure 4 illustrates how our proposition may account for the recent results obtained by Jadhav et al. (2012). In this study, rats experienced a W-track spatial alternation task: they alternated between “inbound” trials where they had to go to the center starting from either the left or the right arm and “outbound” trials where they had to go from the central arm to the arm (left or right) that they did not visit on the previous trial (Figure 4A). Outbound trials present a higher degree of difficulty in that they require linking past experience—the previously experienced side of the maze—with current location in order to make an appropriate decision. Strikingly, lesion of the hippocampus impaired both inbound and outbound learning (Kim and Frank, 2009) while disruption of awake hippocampal replay only impaired outbound learning (Jadhav et al., 2012).

We show on Figure 4B (resp. C) how a model-free (resp. model-based) system dependent on hippocampal input could explain the results. A model-free system learning the association between a spatial state (i.e., left arm, right arm, or central arm) and an action would be able to learn inbound trials but not outbound trials. This is because the “center” state is half of the time followed by rewarded trials on the left and half of the time followed by rewarded trials on the right, thus producing a situation with high uncertainty. In contrast, a model-based system learning to associate previous state transitions with actions can solve both inbound and outbound trials (Figure 4C). Thus, within our proposal, hippocampal lesions impair both inbound and outbound learning because they suppress spatial information required by both place-based model-free and model-based systems. By contrast, disruption of hippocampal awake replay would impair only the model-based system, potentially by blocking the storage of transitions in the model (Gupta et al., 2010), sparing the model-free system to still learn inbound trials.

## 5. Ventral striatum—model builder?

What, then, might be the role of the VS in model-free and model-based navigation? Ventral striatal recordings and lesion studies have provided strong evidence for an evaluative role, either as part of the “critic” contributing to the calculation of the reward prediction error (O'Doherty et al., 2004; Khamassi et al., 2008), or as the locus for general Pavlovian-instrumental transfer where rewarded stimuli act to motivate future action (Corbit et al., 2001; Yin et al., 2008; Corbit and Balleine, 2011). The actor/critic architecture is a variant of the model-free reinforcement algorithms, which conceptually splits the value learning and action selection components (Sutton and Barto, 1998): the critic learns the value of every state, and uses those values to compute the reward prediction error after each state transition *s* to *s*′, given any reward obtained; the prediction error is used by the actor to change the probability of selecting each action in state *s*, thus reflecting the outcome. The existing evidence that dorsal striatum supports action selection while the VS supports stimulus-outcome association has led to proposals that they respectively subserve the actor and critic roles (Joel et al., 2002; O'Doherty et al., 2004; Khamassi et al., 2005, 2008; Daw et al., 2011; van der Meer and Redish, 2011). The primary candidate for transmitting the reward prediction error is the phasic activity of the midbrain dopamine neurons (Schultz et al., 1997; Bayer and Glimcher, 2005; Cohen et al., 2012); further strengthening the proposed identification of the VS with the critic is that it is the major source of inputs to the dopamine neurons (Watabe-Uchida et al., 2012) that in turn project to the dorsal striatum (Maurin et al., 1999; Haber et al., 2000) (see Figure 6).

We sketch an account here that finesses this view, extending previous proposals (Yin et al., 2008; Bornstein and Daw, 2011) for separately considering the core and shell. We first argue that in addition to being useful for the “critic” in model-free processes, reward information encoded by the VS also contributes to model-based processes such as the building of a reward function. Second, from the perspective of navigation tasks, we find evidence that the core of the VS is a key locus for learning the correct sequences of actions in a task. A useful consequence of considering this proposed model-based/model-free dichotomy in both conditioning and navigation is that, whereas the core of the VS is often ascribed a purely evaluative role in the conditioning literature (Yin and Knowlton, 2006; Yin et al., 2008; Bornstein and Daw, 2011), the literature on core involvement in navigation clearly points to a major role in the direct control of locomotion. For the shell of the VS, we discuss further the suggestion that it is a key locus of the critic that signals the reward prediction error for the model-based system (Bornstein and Daw, 2011)^5^; we also discuss the possibility that it acts as a critic that signals a *state* prediction error in the predicted and actual state transitions. As these functions of the core and shell are essential for correct assemblage of the “model” of the world, we informally label the VS as part of the “model-builder”.

### 5.1. Ventral striatum as substrate for building the reward function

In the machine learning literature, one of the requirements for model-based algorithms is to build the so-called “reward function” which relates states to rewards [see Figure 1; (Sutton and Barto, 1998)]. In spatial tasks, this consists of memorizing the places in which reward is found. This is crucial information for deliberative decision-making where inference of future outcomes within the estimated world model—e.g., the tree-search process—requires reaching a terminal state where a reward can be found. The reward function is also important for off-line simulations within the world model to consolidate trajectories leading to reward—see for instance the *DynaQ* algorithm (Sutton and Barto, 1998). Indeed, such mental simulations should be informed when the agent has virtually reached a state containing a reward, although the agent is not necessarily physically experiencing such reward.

Interestingly, sequences of hippocampal place cell activations that occur while an animal is running a track in search for reward are known to be replayed during subsequent sleep (Euston et al., 2007) or during awake resting periods (Foster and Wilson, 2006; Gupta et al., 2010). These replay events have been hypothesized to participate in the consolidation of relevant behavioral sequences that lead to reward. Of particular interest for this review are recent reports of off-line synchronous replay between ventral striatal and hippocampal activity (Lansink et al., 2009). Lansink et al. (2009) found pairs of hippocampus—VS neurons that were reactivated during awake fast forward replay preferentially if: the hippocampal cell coded for space, the ventral striatal cell coded for reward, and the hippocampal cell was activated slightly before the ventral striatal cell during the task. The reactivation occurred 10 times faster than the sequence of activity during the task execution, possibly complying with physiologically plausible eligibility timing. The ventral striatal cells were predominantly in the core—but also included the shell. By illustrating possible neural mechanisms for the off-line consolidation of place-reward associations, these results provide striking examples of activity that could underly the building of the “reward function”, which relates states to rewards.

Of course, it is plausible that such replay events could at the same time be used to update value estimations and action probabilities in the model-free system, consistent with the hypothesized *critic* role of part of the VS (O'Doherty et al., 2004; Khamassi et al., 2008; Bornstein and Daw, 2011). But if the ventral striatal part engaged during these replay events was only dedicated to model-free reinforcement learning, all ventral striatal cells encoding reward predictions in any location—not only in the reward location—should be reactivated in correspondence with the hippocampal cells coding for their associated states, which is not the case here. These results thus emphasize that the VS's evaluative role and its involvement in encoding reward information may also contribute to model-based processes. In support of this view, McDannald et al. (2011) recently showed in rats experiencing an unblocking procedure that VS not only incorporates information about reward value but also about specific features of the expected outcomes. Along with the orbitofrontal cortex, VS was indeed found to be required for learning driven by changes in reward identity, information only relevant for model-based processes but not for model-free ones which only work with value information.

Now where does the information which is replayed off-line between VS and hippocampus come from? One possibility is that relevant place-reward associations experienced during task performance are tagged in order to be preferentially replayed during subsequent sleep or awake resting periods. In support of this proposition, van der Meer and Redish (2010)'s synchronous recordings of VS and hippocampus in a T-maze disentangled possible mechanisms underlying the binding of hippocampal place representations and ventral striatal reward information during task performance. They found a ventral striatal phase precession relative to the hippocampal theta rhythm. This phase precession was found in ventral striatal ramp neurons preferentially receiving input from those hippocampal neurons that were active leading up to reward sites. This phenomenon was accompanied by increased theta coherence between VS and the hippocampus, possibly underlying the storage of relevant place-reward associations that should be tagged for subsequent consolidation.

### 5.2. Ventral striatal core as substrate for building the action model

Yin et al. (2008) proposed that one of the core's primary functions is to learn stimulus-outcome associations that drive preparatory behavior such as approach. Bornstein and Daw (2011) proposed in turn that, as preparatory behavior is value-agnostic, this is consistent with the core playing the role of the critic in a model-free controller: that it either computes directly or conveys the values of current and reached state to midbrain dopamine neurons (Joel et al., 2002), which in turn signal the reward prediction error to targets in the striatum and PFC (Schultz et al., 1997; Dayan and Niv, 2008). This proposal naturally extends to the core playing the role of model-free critic in navigation as well as conditioning.

However, it is equally clear that the core has a role in direct control of motor behavior, and may even serve as an action selection substrate separate from the dorsal striatum (see Pennartz et al., 1994; Nicola, 2007; Humphries and Prescott, 2010 for reviews). These dual roles for the core are not in conflict: the separate populations of core neurons that either project to the dopaminergic neurons of the midbrain or project to the other structures of the basal ganglia could, respectively, fulfill the evaluative and motor control roles (Humphries and Prescott, 2010). Here we focus on how the latter role may fit into a putative model-based/model-free separation of navigation based on the dorsal striatum.

It has long been known that core application of NMDA, AMPA, or dopamine agonists, or of drugs of abuse (amphetamine, cocaine), induces hyperlocomotion in rats, and that intact output of the core through the basal ganglia is necessary for this hyperlocomotion to occur (Pennartz et al., 1994; Humphries and Prescott, 2010). The phasic activity of individual core neurons also correlates with the onset of locomotion during self-administration of cocaine (Peoples et al., 1998). During behavioral tasks, the activity of individual neurons in the core correlates with the direction of upcoming movement, irrespective of the properties of the cue used to prompt that movement (Setlow et al., 2003; Taha et al., 2007). Moreover, when rats navigate a maze, the activity of core neurons correlates with the direction of movement in specific locations (Shibata et al., 2001; Mulder et al., 2004). Together, these data suggest that the core not only directly controls movement, but also receives spatial information on which to base that control.

In addition, the core is necessary for correctly learning sequences of motor behaviors. Blocking NMDA receptors in the core, which putatively prevents synaptic plasticity, degrades performance on many spatial tasks: rats cannot learn paths to rewards (Kelley, 1999), learn spatial sequences (in this case, of lever presses) to achieve reward (Bauter et al., 2003), or locate a hidden platform in a Morris water maze when encoded by distal cues alone (Sargolini et al., 2003). Lesioning hippocampal afferents to VS by cutting the fornix/fimbra pathway results in numerous spatial navigation problems. Whishaw and colleagues have shown that rats with such lesions have intact place responses, but great difficulty in constructing paths to them (Whishaw et al., 1995; Gorny et al., 2002). In a Morris water maze, lesioned rats can swim to a pre-lesion submerged platform location, but not to a new one (Whishaw et al., 1995); in open-field exploration, lesioned rats do not show path integration trips to their homebase (Gorny et al., 2002). Data from these studies has to be interpreted with care, but are consistent with the NMDA blockade studies. Together these data point to a key role for ventral striatal core in linking together sequential episodes of behavior.

So what is the motor control part of the core doing within the model-based/model-free framework? A general proposition is that the core is the route via which hippocampal sequencing of states reaches the motor system, a finessing of the long-recognized position of the core at the limbic-motor interface (Mogenson et al., 1980). We sketch a proposal here that its specific computational role is to learn and represent the probability of action selection within the transition model of the model-based system.

#### 5.2.1. Actions in the transition model

Consider the transition model *T*(*s′*, *a*, *s*), giving the probability of arriving in state *s*′ given action *a* and current state *s*; which we can also write *p*(*s′*|*a*, *s*). The model has two uses: for off-line learning, it is used to sample trajectories through the world model, and update the values of each state accordingly (Sutton and Barto, 1998; Johnson and Redish, 2005); for on-line action selection, it can be queried for the probability that each action will lead to the desired transition from state *s* to *s*′. To achieve this dual use it might be advantageous to decompose the transition model *p*(*s′*|*a*, *s*) using Bayes theorem into representations of the state transitions and of the probability of action selection:

$$p\left(s\prime |a,\;s\right)=p\left(s\prime |s\right)\frac{p\left(a|s\prime ,\;s\right)}{p\left(a|s\right)},$$

where we assume that current state *s* is known. The first-term *p*(*s′*|*s*) is then just the probability model for state transitions, the second term is just the probability *p*(*a*|*s′*, *s*) that each action will cause that transition, normalized by the probability *p*(a|s) of ever taking that action in state *s*. Consequently, off-line learning is a product of the two terms, whereas on-line action selection can be based on the second term only.

Such a decomposition in turn suggests a decomposition into neural substrates. The hippocampal formation has long been proposed to represent potential state transitions (Poucet et al., 2004), and so is a natural candidate for representing *p*(*s′*|*s*) in the simultaneous activity of current (*s*) and adjacent (*s*′) place cells. Alternatively, neural network modeling of hippocampal formation functions in spatial navigation has even suggested that the directional-specificity of many place fields could be interpreted not as place cells but rather as “transition” cells, representing the possible transitions between the current and next “states” in the environment (Gaussier et al., 2002). In this account, each cell is a candidate for directly encoding *p*(*s′*|*s*).

The ventral striatal core is then a potential substrate for representing the transition-conditioned probability of action selection *p*(*a*| *s′*, *s*). A plausible network implementation is that hippocampal outputs representing *s* and *s*′ converge on neuron groups in the core, whose consequent activity is then proportional to *p*(*a*|*s′*, *s*). Learning this action component *p*(*a*|*s′*, *s*) of the transition model is then equivalent to changes in the synaptic weights linking the two state representations in hippocampus to the neuron group in the core. Over all known state transitions from the current state *s*, the activity in the core then encodes a probability distribution over potential actions; the selection of action based on this distribution is then done by the core's corresponding basal ganglia circuit (see Redgrave et al., 1999; Nicola, 2007; Humphries and Prescott, 2010; Humphries et al., 2012 for detailed models of this process).

This decomposition into substrates suggests that core neurons should thus show activity correlated with both off-line model search and on-line action selection. The latter we have already discussed: core activity is correlated with specific actions; in particular, the studies of Shibata et al. (2001) and Mulder et al. (2004) showing a set of core neurons with motor-related activity only in specific places within a maze (such as an arm), and then only when the rats move in a particular direction in that place (e.g., toward the arm end), are consistent with the encoding of action probability conditioned on a transition between states. This substrate decomposition also suggests that hippocampal formation and the core should be synchronized throughout free exploration, as continually changing states represented in hippocampus should have a corresponding recruitment of changing action selection probabilities in the core—just such an exploration-specific synchronization in local-field potentials between hippocampus and the core has been reported by Gruber et al. (2009). More electrophysiological studies will be required to confirm this hypothesis and precisely identify the underlying mechanisms.

Recent neurophysiological studies also support the existence of neural activity consistent with off-line model use for decision-making in the core. In a multiple T-maze, van der Meer and Redish (2009) found that neurons in the core which fired at either reward site also fired at the maze's decision point, just where hippocampal activity correlates of forward planning have been previously found (Johnson and Redish, 2007). Such activity at decision points occurred before reward was actually experienced, and thus before error correction. This activity appeared only during initial stages and disappeared after additional training producing behavioral automation. Such activity could thus reflect a search process related to the early use of model-based processes for decision-making by providing signals for the evaluation of internally generated possible transitions considered during navigation (van der Meer and Redish, 2009).

### 5.3. Ventral striatal shell as critic(s) in the model-builder: one system amongst many

More than any other region of the striatum, the ventral striatal shell is a complex intermingling of multiple separate systems (Humphries and Prescott, 2010), which may include control of approach and aversive behaviors (Reynolds and Berridge, 2003), hedonic information, outcome evaluation, memory consolidation, and appetitive control (Kelley, 1999). Consequently, we cannot meaningfully speak of *a* role for the shell; not least because, as we noted in Humphries and Prescott (2010), the lateral and medial shell are themselves easily distinguished entities in terms of their afferent and efferent structures—we will return to this distinction below.

Yin et al. (2008) proposed that the shell's primary function is to learn stimulus-outcome associations that drive consummatory behavior. Bornstein and Daw (2011) argued that this role in consummatory behavior requires a sensitivity to the values of the outcome, and thus makes the shell a natural candidate for subserving a role equivalent to the “critic” for the model-based system. While strictly speaking the actor/critic algorithm is a model-free system, the model-based system still may rely on the computation of a prediction error to update the values of each state (van der Meer and Redish, 2011), whether during off-line model search or on-line update after each performed action. Recently, Daw et al. (2011) tested human subjects on a multi-stage decision task that separated model-based and model-free prediction errors, and found that the model-based prediction error correlated with the fMRI BOLD signal in VS.

Against this idea, earlier work has shown that the shell appears not to be required for knowledge of the contingency between instrumental actions and their outcomes: lesioning the shell does not stop devaluation or contingency changes from changing behavioral choice (Balleine and Killcross, 1994; Corbit et al., 2001). Consequently, the shell could appear not to be necessary for establishing goal-directed learning—or, by extension, model-based learning.

However, a closer reading of the lesion studies allows us to refine that conclusion. In “shell” lesion studies, only the medial shell is targeted (see, for example, Figure 1 of Corbit et al., 2001)—not a flaw in experimental design but a limitation imposed by anatomy, as attempts to lesion the lateral shell would undoubtedly also damage the overlying lateral core (Ikemoto, 2002). Consequently, the lateral shell remains intact, and is thus a prime candidate for a model-based critic that leaves the animal sensitive to outcome devaluation and contingency changes.

**Figure 5:**
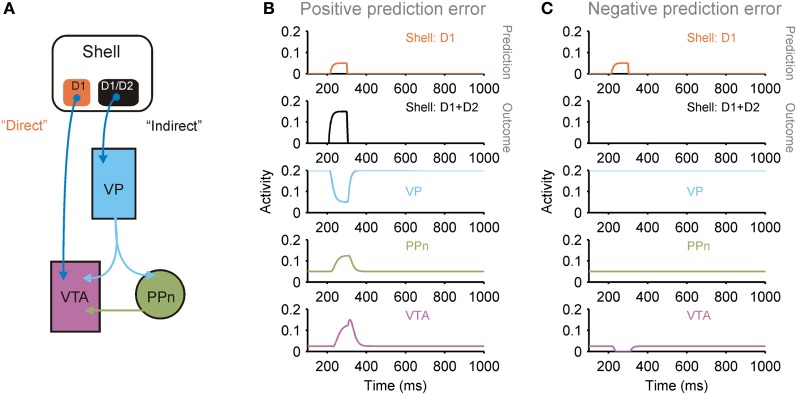
**Dual pathways from shell to ventral tegmental area (VTA) potentially support prediction error computation. (A)** The medial and lateral shell both support a dual pathway circuit that converges on dopaminergic neurons in the VTA: a direct pathway originating from a population of D1 receptor expressing striatal projection neurons, and an indirect pathway originating from a mixed population of D1 and D2 receptor expressing striatal projection neurons [see (Humphries and Prescott, 2010) for review]. This arrangement is consistent with the shell's role as a “critic”: the pathways support the computation of a prediction error between the prediction transmitted by the direct pathway and the actual outcome transmitted by the indirect pathway (PPn, pedunculopontine nucleus; VP, ventral pallidum). **(B)** Simulation of neural population activity showing how a greater outcome (indirect pathway) than predicted (direct pathway) drives a phasic increase in VTA activity, signaling a positive prediction error. **(C)** Simulation of neural population activity showing how a lower outcome (indirect pathway) than predicted (direct pathway) drives a phasic dip in VTA activity, signaling a negative prediction error. Simulation details given in Humphries and Prescott (2010).

Moreover, as we detailed in Humphries and Prescott (2010), lateral and medial shell are separable entities: medial shell receives extensive input from hippocampal field CA1 and subiculum, while lateral shell receives scant hippocampal input; and both have separate “direct” and “indirect” pathways through the basal ganglia to separate populations of midbrain dopaminergic neurons (Figure 5A). As we show in Figures 5B,C, the dual pathways are a plausible candidate for computing a prediction error based on comparing the forebrain inputs to the two pathways; consequently both medial and lateral shell could support different “critic” roles (Humphries and Prescott, 2010).

Which leaves the question of the role of the medial shell, if it is indeed in a position to compute a prediction error. In Humphries and Prescott (2010) we proposed the idea that the projections from hippocampal formation and PFC to the “direct” and “indirect” pathways could, respectively, represent the expected and achieved state after a transition. Consequently, the medial shell would be in a position to compute a *state* prediction error, that adjusts the transition probability *p*(*s′*|*s*) based on model predictions, rather than on simply counting the occurrences of each transition.

Lesioning the medial shell would then be predicted to show subtle deficits in tasks that require building a world model: in sufficiently simple tasks, the mere construction of the links between a limited number of states, whose values are correctly learnt, may be sufficient to solve the task and respond to subsequent changes in the value of those states. Consequently, the intact sensitivity to devaluation by medial shell-lesioned rats (Balleine and Killcross, 1994; Corbit et al., 2001) suggests that these were sufficiently simple tasks. That task complexity is a factor is suggested by the data of Albertin et al. (2000). They trained rats on a plus-maze on which a currently lit arm-end contained reward in the form of water drops; each day the rats experienced a new sequence of lit arms, and each day one of the arms was chosen to contain six drops and the others contained one drop. A probe trial was then run in which every arm was lit, allowing the rat to choose which arm to visit. Albertin et al. (2000) found that lesioning the medial shell prevented rats from correctly remembering which maze arm contained the high value reward on a probe trial, but did not impair their ability to learn to visit the lit arm in the sequence during training. Such a task plausibly requires each day building anew a world model and querying it on the probe trial to recall which available state-transition contained the high reward on that day. If damage to the medial shell prevented correct learning of the transition model, then this would selectively impair querying of the model, while leaving intact the ability to do simple light-reward association in the model-free system.

Glascher et al. (2010) searched for correlates of a state prediction error in the fMRI BOLD signal recorded from humans learning a decision-tree of stimulus choices in the absence of reward, which was subsequently used as the basis for a rewarded task. Encouragingly, subjects' behavior during the learning stage was well-fit by a reinforcement learning model incorporating a state prediction error; moreover, the BOLD signal in lateral PFC and intra-parietal sulcus correlated with the state prediction error in the model. The equivalent regions in rat are known afferents of the shell (Uylings et al., 2003; Humphries and Prescott, 2010). However, they reported that the ventral striatal BOLD signal correlated only with the fitted model-free reward prediction error during the rewarded task stage, and not the state prediction error. It is not clear, though, whether something computed by a set of neurons as small as the proposed sub-set in medial shell could be resolved by the voxel-size used, a problem compounded by the conservative multiple-comparison corrections used in searching for BOLD signal correlates.

## 6. Conclusions

**Figure 6:**
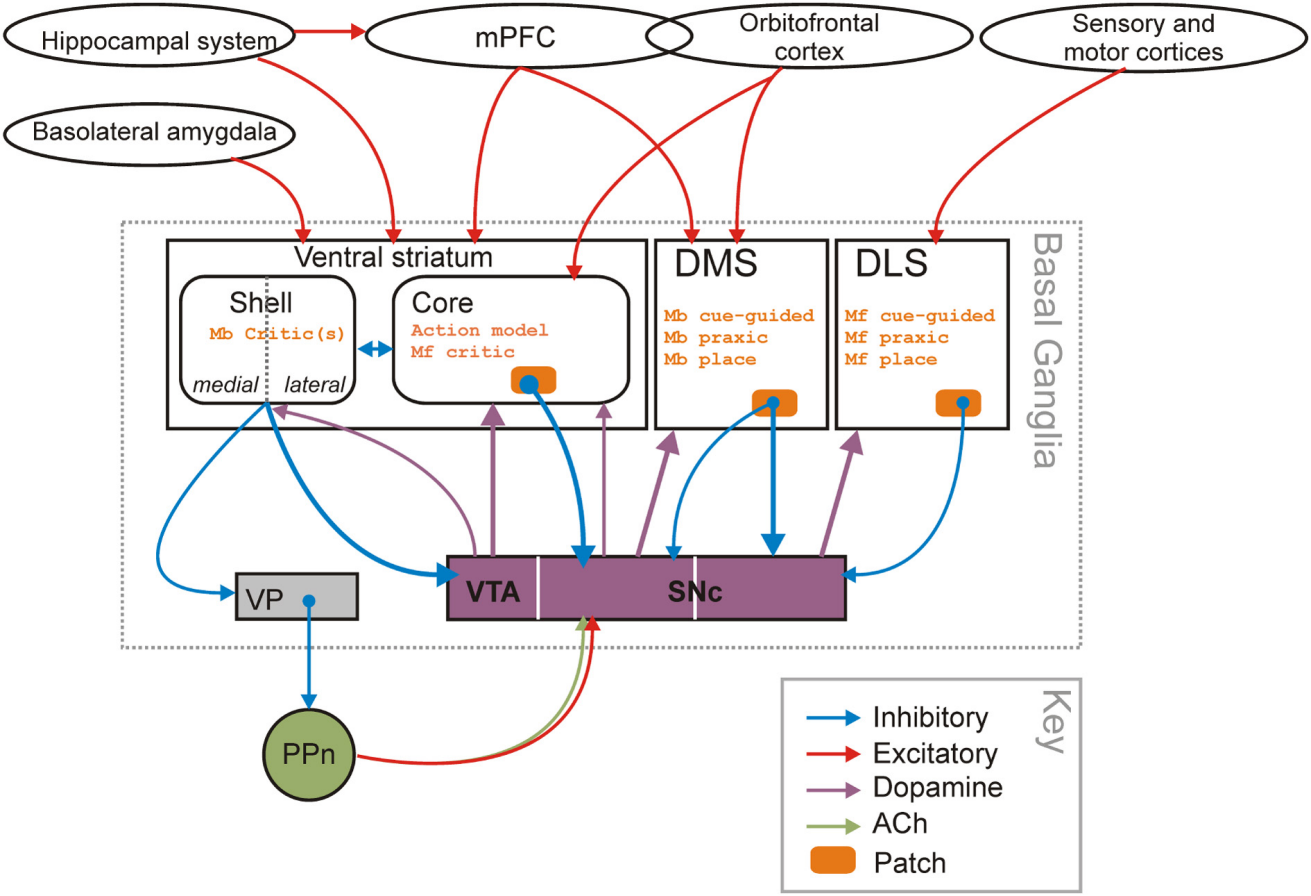
**Striatal-domain substrates of model-free and model-based controllers.** The proposed organization of navigation strategies and potential control of learning across the three striatal domains. The identification of the shell and core as “critics” for the model-based and model-free controllers in dorsal striatum partly rests on the “spiral” of striatal-dopamine-striatal projections (Maurin et al., 1999; Haber et al., 2000; Haber, 2003), originating in the shell of the VS (the spiral is indicated by the thicker lines) and on the permissive role dopamine plays in plasticity at cortico-striatal synapses (Reynolds et al., 2001; Shen et al., 2008). There are also closed loop links between dopamine cell populations and each striatal region. Abbreviations: Mb, model-based; Mf, model-free; PPn, pedunculopontine nucleus; SNc, substantia nigra pars compacta; VP, ventral pallidum; VTA, ventral tegmental area. Note that the “inhibitory” and “excitatory” labels refer to the dominant neurotransmitter of the connection, not the effect that connection may have on the target nucleus as a whole (e.g., basolateral amygdala input to VS neurons can suppress other excitatory inputs despite using glutamate, which is an “excitatory” neurotransmitter).

In this paper, we have proposed a functional distinction between parts of the striatum by bridging data about their respective involvement in behavioral adaptation taken from both the spatial navigation literature and the instrumental conditioning literature. To do so, we have first formally mapped taxonomies of behavioral strategies from the two literatures to highlight that navigation strategies could be relevantly categorized as either model-based or model-free. At root, the key distinction is that it is the *use* of information in building a world representation, rather than the *type* of information (i.e., place vs. cue), that defines the different computational processes at stake and their substrates in the striatum. Within this framework, we explicitly identified the role for dorsolateral striatum in learning and expression of model-free strategies, the role of dorsomedial striatum in learning and expression of model-based strategies, and the role of “model-builder” for the VS—most probably in conjunction with the hippocampus (Lansink et al., 2009; van der Meer et al., 2010; Bornstein and Daw, 2012). Our scheme is summarized in Figure 6.

The hypothesis that two decision-making systems (i.e., model-based and model-free) are processed in parallel in DMS and DLS while VS is important for the acquisition of the model seems to well explain the results of Atallah et al. (2007). In a forced-choice task in a Y-maze requiring rats to learn the association between two odors and two actions (go left or right), they found that transient inactivation of DLS^6^ did not prevent a covert learning process which became visible as soon as the DLS was released. Although this task is typically interpreted as a habit learning task (van der Meer et al., 2012), the absence of over-training in the animals—60 trials performed in total—suggests that model-based learning in the DMS was still playing an important role at this stage and was unaffected by DLS inactivation. Moreover, Atallah et al. (2007) found that inactivation of VS mostly impaired acquisition and only partially affected performance, consistent with the proposed role of VS in building the model used by the model-based system.

### 6.1. Computations by the striatum

Our proposed division of function between different parts of the striatum preserves the classical hypothesis that striatal territories all contribute to behavioral regulation but mainly differ in function because of their different afferents (Alexander et al., 1990; Joel and Weiner, 1994; Middleton and Strick, 2000)—a common division of cortical afferents among the striatal territories is illustrated in Figure 6. Throughout its dorso-lateral to ventro-medial extent, the striatum has a consistent micro-circuit dominated by GABAergic projection neurons controlled by at least three classes of interneurons (Tepper et al., 2004; Bolam et al., 2006; Humphries and Prescott, 2010). Such a consistent micro-architecture points to common operational principles for how striatum computes with its afferent inputs. Moreover, the cortex-basal ganglia-thalamus-cortex anatomical loop involving the ventral striatal core respects the same organization principles as loops involving the dorsal striatum: thus DLS, DMS, and VS core are all involved in complete basal ganglia circuits composed of direct and indirect pathways (Humphries and Prescott, 2010). Since numerous computational studies have shown that this basal ganglia circuitry is efficient for performing a selection process (Houk and Wise, 1995; Mink, 1996; Redgrave et al., 1999; Humphries et al., 2006; Leblois et al., 2006; Girard et al., 2008), it has been proposed that loops involving different striatal territories could perform different levels of selection influencing behavior. One such scheme envisions a hierarchy running from course-grained selection of overall goal or strategy to achieve a goal, through actions toward a goal, to fine-grained movement parameters of each action (Redgrave et al., 1999; Ito and Doya, 2011).

The model-based/model-free dichotomy would respect such a general principle of common selection operation: that striatal territories receiving state transition information (i.e., *p*(*s′*|*s*) corresponding to the probability of transition from state *s* to state *s*′, no matter if these states are spatial or determined by a perceptual cue) would be involved in model-based action selection while striatal territories receiving simple state information (i.e., *p*(s), no matter if state *s* represents a spatial position or the perception of a stimulus) would be involved in model-free action selection. As we discussed throughout the text, in contrast to DLS, VS and DMS receive direct projections from the hippocampal system as well as medial PFC which place them in a good situation to process hippocampal state transition information (Gaussier et al., 2002; Poucet et al., 2004) and hence to participate in the model-based action selection. Correspondingly, the dominant projections of sensorimotor cortices to DLS may thus convey current state information, whether originating from the periphery or from higher cortical areas (Haber, 2003), and hence the DLS participates in model-free action selection.

### 6.2. Open questions

The account here provides concrete proposals for the dorsolateral and dorsomedial striatum's role in spatial navigation, while introducing new but comparatively speculative ideas about the VS's roles in the model-free and model-based systems. As such, our account is of course incomplete; so let us conclude with the primary open questions:

- We have drawn a distinction between place/response strategies and model-based/model-free use of those strategies. To the best of our knowledge, we lack good evidence for the existence of a model-free place strategy.
- The observations of a place-to-response strategy shift with over-training (Dickinson, 1980; Packard and McGaugh, 1996; Pearce et al., 1998; Chang and Gold, 2003) underpinned the existing idea that a response strategy is by nature habitual. Our hypothesis postulates that the central mechanism underlying all these observed behavioral shifts is a shift from model-based to model-free rather than from place-based to either cue-guided or praxic behaviors; but why then is the shift often (but not always Yin and Knowlton, 2004; Botreau and Gisquet-Verrier, 2010) from model-based place to model-free response?
- What is anterior DMS doing? Ragozzino and Choi (2004) proposed a role for it in strategy selection, as lesions caused a selective deficit in reversal learning, but not in initial acquisition. Alternatively, perhaps DMS is divided into sub-territories differentially involved in place, cue, and praxic model-based systems.
- Lesion data on the core provide conflicting accounts of its roles. For example, the results of Corbit et al. (2001) disagree with evaluation: for why, if the core forms part of the transition model, does lesioning it not then prevent outcome devaluation from affecting behavior? By contrast, McDannald et al. (2011) found that lesions of core affected responding to both changes in outcome value and changes in outcome identity, emphasizing its involvement in model-based learning. From our account, it is not surprising that conflicting data arise if core lesions interfere with both evaluative and action selection systems; however, it is not clear what task designs would be sufficient to tease apart the selective effects of core lesions on its evaluative and action selection roles.
- Do the striatal domains underpin a common computation? Our focus has been on the algorithmic-level distinctions between behavioral strategies, and the striatal substrates within the neural systems implementing those algorithms. As noted throughout, this computation may be action selection: the resolution of competing inputs at the striatal level into one (or a few) selected signals at the output of the basal ganglia. Based on our proposals here, we may speculate that these selections are based on different representations of the world.

### Conflict of interest statement

The authors declare that the research was conducted in the absence of any commercial or financial relationships that could be construed as a potential conflict of interest.

## Footnotes

^1^We use VS throughout, rather than *nucleus accumbens*, to emphasize the contiguous nature of the striatum through its dorsolateral to ventro-medial extent (Voorn et al., 2004; Humphries and Prescott, 2010).

^2^*praxic* normally refers to internally-generated sequences of movement independent of position information.

^3^This fiber pathway brings hippocampal information to the VS, but is also the source of brainstem inputs to the hippocampus, so may disrupt either transmission of place information by hippocampus or the encoding of place in hippocampus.

^4^They used a model-based algorithm that explicitly computed the transition matrix. It seems feasible that simpler model-based algorithms, without explicit computation of the transition matrix, could also equally account for the sensitivity to devaluation and contingency changes in goal-directed learning, as their repeated internal simulation after such outcome manipulations would result in more rapid changes in overt behavior. To our knowledge, no one has examined the possibility. Intriguingly, Johnson and Redish (2005) showed that such an internal-simulation model, emulating hippocampal replay of previous trajectories through a maze, could indeed reduce the onset of habit-like stereotypy in the paths taken through the maze.

^5^This relates to the notion, in the machine learning literature, that some model-based algorithms such as *Dyna-Q* can update their state-action values through a reward prediction error (RPE), although other model-based algorithms based on so-called *value iteration* processes do not rely on a RPE: they instead propagate value information from each state to other proximal states (Sutton and Barto, 1998).

^6^Although the injection site was referred to as the central part of the dorsal striatum by the authors (see Supplementary Figures 3 and 4 of their original paper), the great majority of injections were located outside the dorsal striatal region receiving projections from the prelimbic cortex [see Figure 3 in Voorn et al. (2004)], and thus outside the zone called dorsomedial striatum and related to goal-directed behaviors and model-based learning [see Figure 1 in Yin et al. (2008) and Figure 1 in Bornstein and Daw (2011)]. Thus, the injections seem to have mostly reached the dorsolateral striatum related to model-free habit learning.
